# Structural Basis for the Enhanced Anti-Diabetic Efficacy of Lobeglitazone on PPARγ

**DOI:** 10.1038/s41598-017-18274-1

**Published:** 2018-01-08

**Authors:** Jun Young Jang, Hwan Bae, Yong Jae Lee, Young Il Choi, Hyun-Jung Kim, Seung Bum Park, Se Won Suh, Sang Wan Kim, Byung Woo Han

**Affiliations:** 1 Research Institute of Pharmaceutical Sciences, College of Pharmacy, Seoul National University, Seoul, 08826 Republic of Korea; 2 Department of Chemistry, College of Natural Sciences, Seoul National University, Seoul, 08826 Republic of Korea; 3 CKD Research Institute, Chong Kun Dang Pharmaceutical Corporation, Yongin, 16995 Republic of Korea; 4 College of Pharmacy, Chung-Ang University, Seoul, 06974 Republic of Korea; 5 College of Medicine, Seoul National University, Seoul, 03080 Republic of Korea

## Abstract

Peroxisome proliferator-activated receptor γ (PPARγ) is a member of the nuclear receptor superfamily. It functions as a ligand-activated transcription factor and plays important roles in the regulation of adipocyte differentiation, insulin resistance, and inflammation. Here, we report the crystal structures of PPARγ in complex with lobeglitazone, a novel PPARγ agonist, and with rosiglitazone for comparison. The thiazolidinedione (TZD) moiety of lobeglitazone occupies the canonical ligand-binding pocket near the activation function-2 (AF-2) helix (i.e., helix H12) in ligand-binding domain as the TZD moiety of rosiglitazone does. However, the elongated *p*-methoxyphenol moiety of lobeglitazone interacts with the hydrophobic pocket near the alternate binding site of PPARγ. The extended interaction of lobeglitazone with the hydrophobic pocket enhances its binding affinity and could affect the cyclin-dependent kinase 5 (Cdk5)-mediated phosphorylation of PPARγ at Ser245 (in PPARγ1 numbering; Ser273 in PPARγ2 numbering). Lobeglitazone inhibited the phosphorylation of PPARγ at Ser245 in a dose-dependent manner and exhibited a better inhibitory effect on Ser245 phosphorylation than rosiglitazone did. Our study provides new structural insights into the PPARγ regulation by TZD drugs and could be useful for the discovery of new PPARγ ligands as an anti-diabetic drug, minimizing known side effects.

## Introduction

Peroxisome proliferator-activated receptors (PPARs) are transcription factors activated by a group of ligands belonging to the thyroid/retinoid family (class II) of nuclear receptors^1^. PPARs regulate the transcription of target genes through heterodimerization with retinoid X receptors (RXRs) and binding to cognate peroxisome proliferator response elements (PPREs)^2^. PPARs, known as “lipid-sensing” receptors, are present in three subtypes (PPARα, PPARγ, and PPARδ/β) and regulate lipid and glucose homeostasis^3^. PPARγ is highly expressed in adipocytes and is also expressed in epithelial cells and macrophages. It plays important roles in adipocyte differentiation, insulin resistance, and atherosclerosis^4^.

PPARγ has a remarkably larger ligand-binding pocket (LBP) than other nuclear receptors^5^ and is known to be activated by a wide range of endogenous ligands and synthetic ligands. Although the physiological ligand of PPARγ has not been clearly elucidated yet, the endogenous PPARγ ligands reported so far include polyunsaturated fatty acids^6^, oxidized fatty acids^7^, nitrated fatty acids^8^, lysophospholipids^9^, and prostanoids such as 15-deoxy-Δ^12,14^-prostaglandin J2^10^. Synthetic ligands of PPARγ can be classified as full agonists, partial agonists, and antagonists. When full agonists bind to PPARγ, the activation function-2 (AF-2) helix (i.e., helix H12) changes its conformation and PPARγ recruits coactivator to induce adipogenesis and insulin sensitization^11,12^. PPARγ is also activated by partial agonists. However, partial agonists stabilize PPARγ ligand-binding domain (LBD) in a distinct manner compared with full agonists. Partial agonists stabilize the β-sheet region (β1, β2, and β4, and helix H3 preferentially, but leave AF-2 helix in a very dynamic state^13^. Antagonists exhibit high affinity but do not activate PPARγ, suggesting that the conformational change in AF-2 helix is not sufficient to allow coactivator binding or AF-2 helix exists in its inactive form^14^.

PPARγ is a good therapeutic target for type 2 diabetes mellitus. When the thiazolidinedione (TZD) class of anti-diabetic drugs was first reported in 1980s^15^, the molecular basis of TZD drugs was unclear. In 1990s, TZD drugs were found to bind to PPARγ^16^, and as mentioned above, various synthetic ligands for PPARγ have been developed so far. Full agonists such as TZD drugs are effective in treating type 2 diabetes mellitus, but adverse effects have been problematic, including fluid retention, edema, bone loss, and weight gain^17^. The mechanism underlying the anti-diabetic effects of TZD drugs and their side effects has not been well understood. Recently, Choi *et al*. reported that the phosphorylation of PPARγ at Ser245 (in PPARγ1 numbering; Ser273 in PPARγ2 numbering) by cyclin-dependent kinase 5 (Cdk5) neither activates nor inhibits general transcriptional activity of PPARγ, but it changes the expression of specific genes such as adiponectin and adipsin that are linked to insulin sensitivity^18^. TZD drugs have been known to strongly regulate both the Cdk5-mediated phosphorylation of PPARγ and the expression of PPARγ target genes^18^. Recent study has also revealed that the alternate binding site extending from the third arm of the known LBP of PPARγ could affect the phosphorylation of PPARγ at Ser245^19^. Thus, many researchers are trying to develop synthetic ligands that selectively inhibit the Cdk5-mediated phosphorylation of PPARγ without classical transcriptional agonism^20,21^.

**Figure 1:**
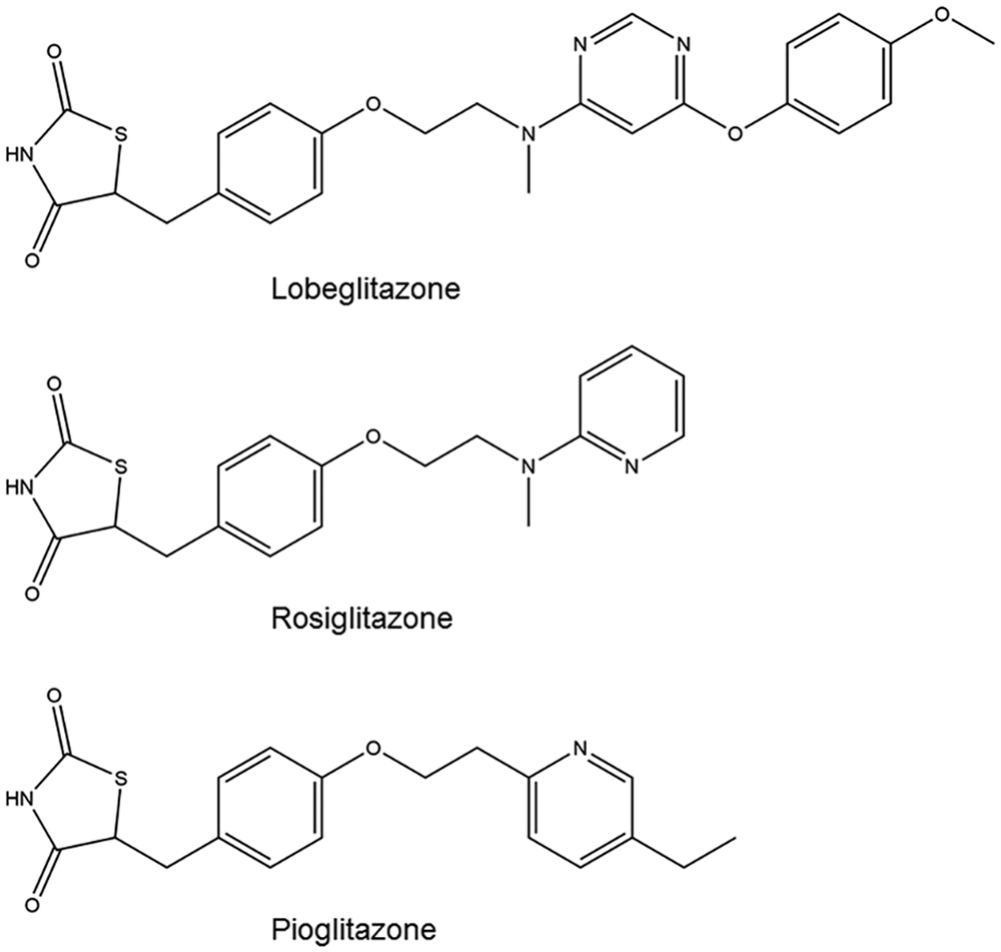
Chemical structures of lobeglitazone, rosiglitazone, and pioglitazone.

Lobeglitazone (CKD-501; Chong Kun Dang Pharmaceutical Corp., Seoul, Republic of Korea) is an anti-diabetic drug of the TZD class known as a potent PPARγ agonist, and has been used in the treatment of type 2 diabetes mellitus^22^. Lobeglitazone is structurally similar to two well-known TZD drugs, rosiglitazone and pioglitazone, and was synthesized from the rosiglitazone backbone by substitution of the pyrimidine moiety for the pyridine part of rosiglitazone followed by addition of the *p*-methoxyphenol functional group at 4-position of the pyrimidine moiety (Fig. 1). Lobeglitazone showed better biological activities than known reference compounds (rosiglitazone and pioglitazone). Compared with rosiglitazone, lobeglitazone showed 100 times increased efficacy in the triglyceride accumulation experiments in 3T3-L1 cells and 2.4-fold and over 8-fold increased efficacies in the glucose and triglyceride lowering experiments in KKA^y^ mice, respectively^23^. To our surprise, rosiglitazone and pioglitazone exhibit different clinical adverse events, which implies that slight changes in receptor-ligand interactions can cause significant pharmacological differences in TZD drugs^13^. Thus, studies of PPARγ-ligand complex structures are crucial to better understand the mechanism of PPARγ modulation and it is also true with a more potent anti-diabetic drug, lobeglitazone. To elucidate a structural basis for understanding activities of lobeglitazone, we determined the crystal structure of PPARγ LBD in complex with lobeglitazone. For exact comparison of binding modes of lobeglitazone and rosiglitazone, we also determined the crystal structure of PPARγ LBD in complex with rosiglitazone. The overall binding mode of lobeglitazone to PPARγ LBD is very similar to that of rosiglitazone except the elongated *p*-methoxyphenol group of lobeglitazone. Notably, the elongated *p*-methoxyphenol group occupies the hydrophobic pocket near the alternate binding site of PPARγ. Lobeglitazone also blocked the Cdk5-mediated phosphorylation of PPARγ at Ser245 more potently than rosiglitazone did *in vitro*. Our results enhance the current understanding of PPARγ regulation by TZD drugs more in detail.

## Results

### Overall structure of lobeglitazone-bound PPARγ LBD and structural comparisons

**Figure 2:**
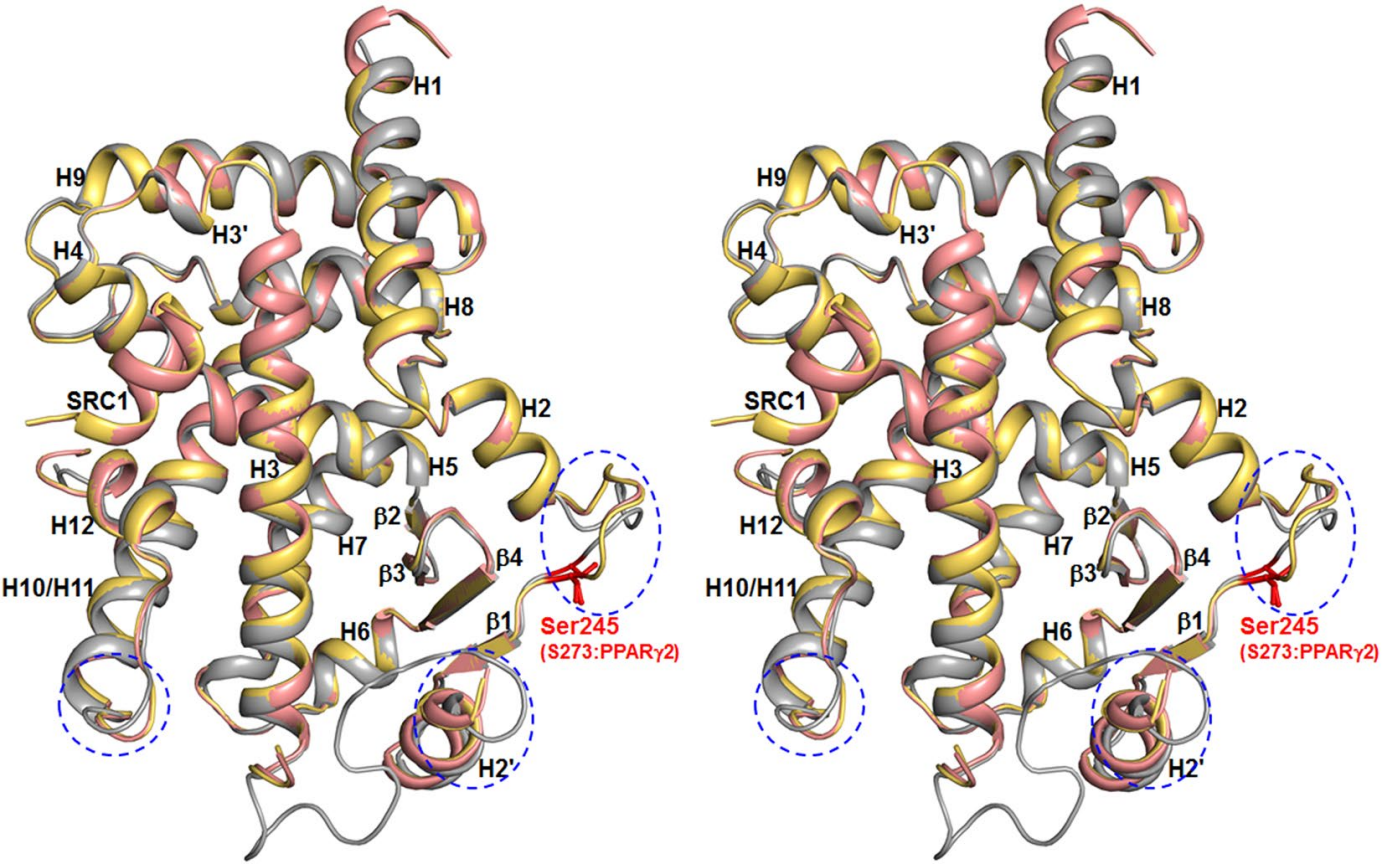
Overall structures of PPARγ LBD in complex with lobeglitazone and with rosiglitazone. Lobeglitazone-bound and rosiglitazone-bound PPARγ LBD structures are superimposed and colored in salmon and yellow orange, respectively, all of which include the SRC-1 coactivator peptide colored same with bound PPARγ LBD structures. Lobeglitazone and rosiglitazone are represented as a stick model in green and pale cyan, respectively. Ser245 (PPARγ1 numbering; Ser273 in PPARγ2 numbering) is the Cdk5-mediated phosphorylation site, shown by red sticks. Regions with RMSD 2.0 Å and bigger are indicated with blue dashed circles. Overall structures are shown in stereo view.

**Figure 3:**
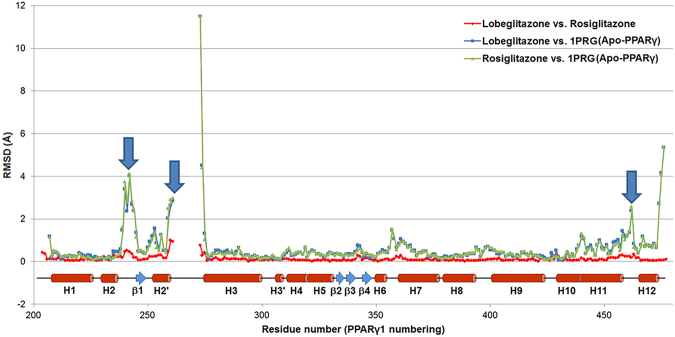
Structural comparison of lobeglitazone-bound, rosiglitazone-bound, and apo PPARγ LBD structures. RMSD values of lobeglitazone-bound vs. rosiglitazone-bound, lobeglitazone-bound vs. apo PPARγ (PDB ID: 1PRG), and rosiglitazone-bound vs. apo PPARγ were calculated and represented in red, blue, and green lines, respectively. Secondary structure elements are indicated on the residue numbers. Regions with RMSD 2.0 Å and bigger are marked with blue down arrows. Residues 262–272 are disordered in the PPARγ LBD structures and were excluded from calculations.

To gain further insight on the binding mode of lobeglitazone, we solved the crystal structure of PPARγ LBD in complex with lobeglitazone in the presence of a peptide derived from the human steroid receptor coactivator-1 (SRC-1) coactivator protein at 2.15 Å resolution, using X-ray crystallography. For a comparative study, we have also determined the rosiglitazone-bound structure of PPARγ LBD in the presence of the SRC-1 coactivator peptide. The structures adopt the canonical fold of general nuclear receptors^1^, comprising one bundle of 13 α-helices and one four-stranded β-sheet (Fig. 2). Helices H10 and H11 of PPARγ LBD forms one helix in our structures, but they were split for the convenience of comparison with other nuclear receptors^24^. The C-terminal helix H12 exists in the active conformation in that it covers LBP of PPARγ. The canonical helical LXXLL motif of the SRC-1 coactivator is stabilized by hydrophobic cleft that is formed by helices H3, H4, H5, and H12 of PPARγ LBD (Fig. 2)^11^. Residues 262–272, belonging to the so-called Ω loop, could not be modeled due to the lack of electron densities. The overall structures of PPARγ LBD in complex with lobeglitazone and with rosiglitazone are similar to the apo PPARγ LBD structure with Protein Data Bank (PDB) ID 1PRG^11^, with root-mean-square deviation (RMSD) of 1.16 Å and 1.17 Å, respectively, for 259 C_α_ atoms (Fig. 3). However, when lobeglitazone or rosiglitazone binds to PPARγ LDB, it induces conformational changes in the H2-β1 loop, Ω loop, and H11-H12 loop compared with the apo PPARγ LBD structure. The structures of lobeglitazone-bound and rosiglitazone-bound PPARγ LBD are almost similar with RMSD of 0.17 Å for 263 C_α_ atoms (Fig. 3).

### Binding mode of the TZD head group and methylamino group in lobeglitazone

**Figure 4:**
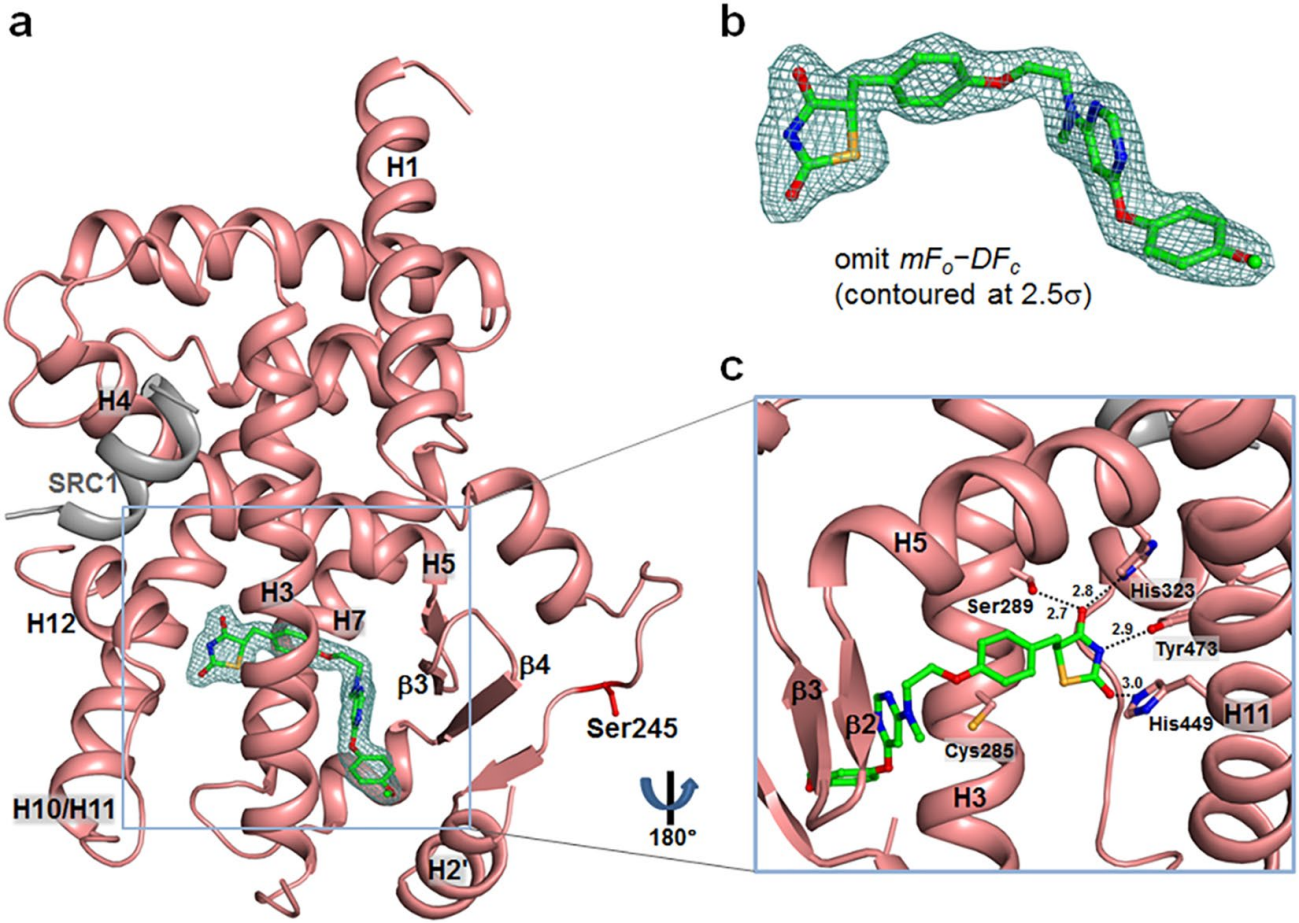
Overall structure of lobeglitazone-bound PPARγ LBD. (**a**) Ribbon diagram of lobeglitazone-bound PPARγ LBD (salmon) with the SRC-1 coactivator peptide (grey). Lobeglitazone shown as a green stick model occupies LBP of PPARγ. The electron density for lobeglitazon in the *mFo-DFc* omit map is shown as a light teal-colored mesh (contoured at 2.5σ). The Cdk5-mediated phosphorylation site, Ser245, is represented by red sticks. (**b**) Close-up view of bound lobeglitazone in sticks with the *mFo-DFc* omit electron density map (contoured at 2.5σ). (**c**) Close-up view of interactions between PPARγ LBD and lobeglitazone. The view is 180° rotated from the orientation in (**a**). Hydrogen bonds are depicted by dashed lines and labeled with donor–acceptor distances in Å. Helix H7 has been omitted to show clear position of lobeglitazone. Cys285 is represented in sticks.

**Figure 5:**
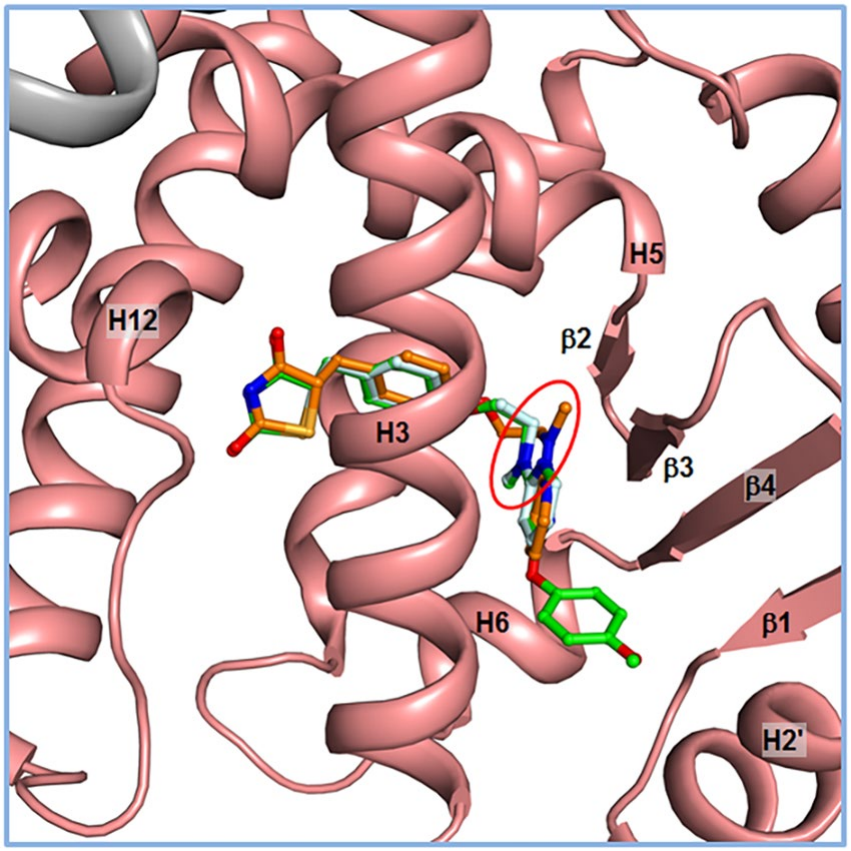
Comparison of binding modes between lobeglitazone and rosiglitazone to PPARγ LBD. The lobeglitazone-bound structure is shown in a ribbon diagram (salmon) and lobeglitazone is represented by a green stick. Rosiglitazone in our structure (pale cyan) is drawn by superimposing the rosiglitazone-bound structure of PPARγ LBD onto the lobeglitazone-bound structure. The representative rosiglitazone-bound PPARγ LBD structure (PDB ID: 2PRG, orange) is also superimposed onto the lobeglitazone-bound structure and rosiglitazone from the representative rosiglitazone-bound PPARγ LBD structure is represented by an orange stick. The red ellipse indicates the methylamino groups.

In the structure of lobeglitazone-bound PPARγ LBD, the ligand omit map calculated from the refined model revealed a clear extra electron density that could be modeled as lobeglitazone later (Fig. 4a and b). Comparison between structures of lobeglitazone-bound and rosiglitazone-bound PPARγ LBD showed that both ligands adopt an U-shaped conformation when bound to PPARγ LBD and wrap around Cys285 on helix H3, resulting in direct contacts with the AF-2 helix (Fig. 4a and Supplementary Fig. S1a). Rosiglitazone-bound structure also showed a clear omit map of rosiglitazone (Supplementary Fig. S1a and b). As shown in Fig. 5, the binding modes of lobeglitazone and rosiglitazone are almost identical up to the sharing chemical moiety. The interactions between the TZD head group of lobeglitazone and PPARγ LBD form three hydrogen bond networks (Fig. 4c). One of two carbonyl groups of lobeglitazone makes a bifurcated hydrogen bond with N_ε_ atom of His323 and O_γ_ atom of Ser289 with hydrogen bond distances of 2.8 Å and 2.7 Å, respectively. The other carbonyl group of lobeglitazone forms a hydrogen bond with N_ε_ atom of His449 on helix H11 with a hydrogen bond distance of 3.0 Å. The partly negatively charged nitrogen of the TZD head group is also stabilized by a hydrogen bond with O_η_ atom of Tyr473 on helix H12 with a hydrogen bond distance of 2.9 Å. Compared with the TZD head group of rosiglitazone, lobeglitazone forms slightly less strong hydrogen bond networks with AF-2 helix of PPARγ LBD (Fig. 4c and Supplementary Fig. S1c). However, when compared with the known representative rosiglitazone-bound PPARγ LBD structure with PDB ID 2PRG^11^, the methylamino group of rosiglitazone in the representative rosiglitazone-bound structure forms a upward conformation with respect to helix H3 while it adopts a downward conformation for lobeglitazone and rosiglitazone in our structures (Fig. 5). This subtle conformational discrepancy in the methylamino group of TZD drugs needs further attention and will be discussed later.

### Binding mode of the *p*-methoxyphenol moiety in lobeglitazone

**Figure 6:**
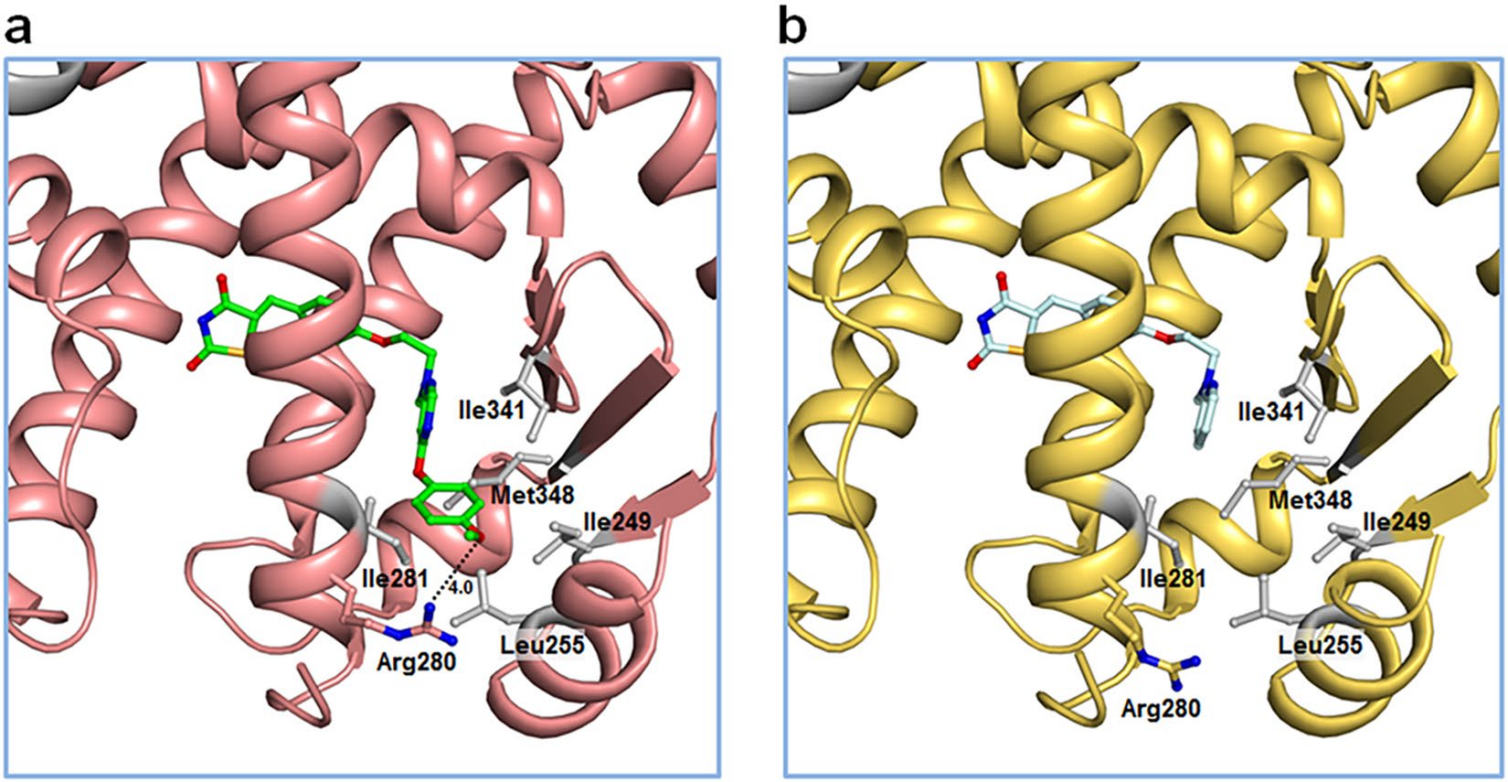
Interactions of the *p*-methoxyphenol group of lobeglitazone with PPARγ LBD. (**a**) The lobeglitazone-bound PPARγ LBD structure is shown in a ribbon diagram (salmon) and lobeglitazone is represented by a green stick. The residues forming the hydrophobic pocket are shown in gray sticks. The *p*-methoxyphenol group of lobeglitazone occupies the hydrophobic pocket. The hydrogen bond between the *p*-methoxyphenol group of lobeglitazone and N_η_ atom of Arg280 is depicted by dashed line and labeled with donor–acceptor distance in Å. (**b**) The rosiglitazone-bound PPARγ LBD structure is shown in a ribbon diagram (yellow orange) and rosiglitazone is represented by a pale cyan stick. The hydrophobic pocket is not occupied. Interaction between rosiglitazone and Arg280 is not observed.

**Figure 7:**
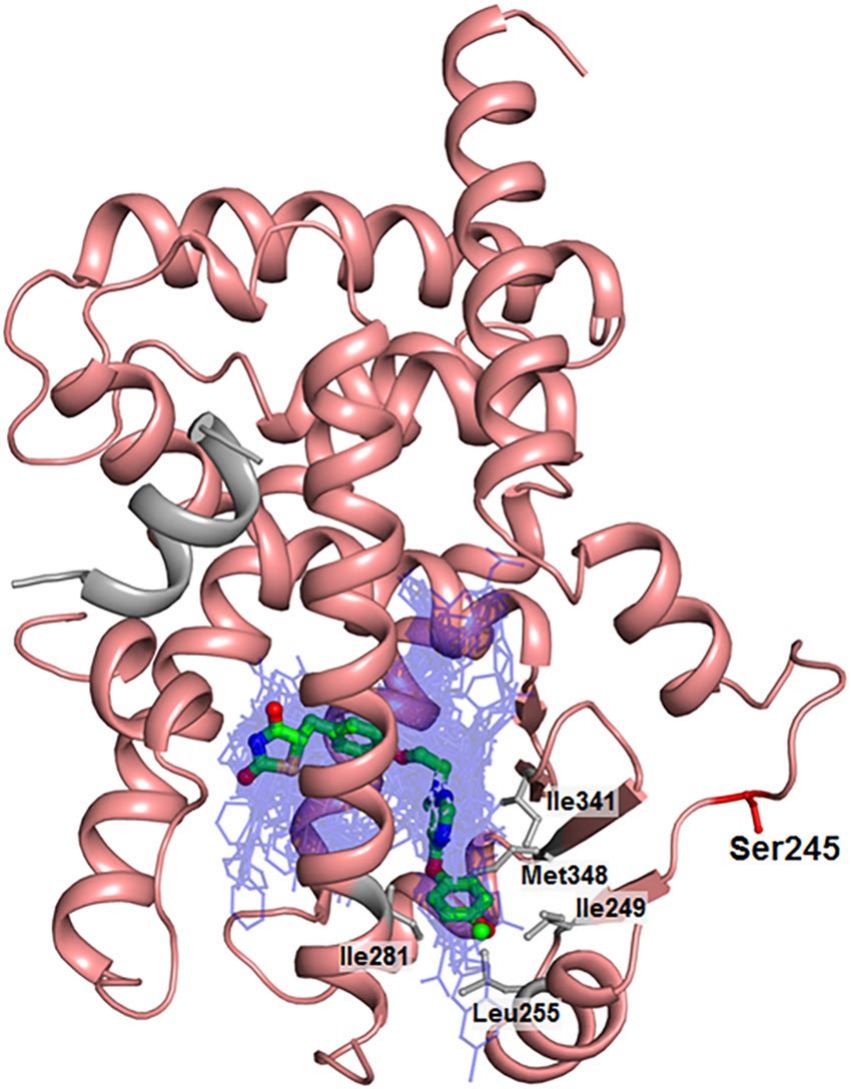
Superposition of lobeglitazone with other PPARγ ligands from known complex structures. 131 PPARγ LBD structures with bound ligand in PDB were superimposed onto the lobeglitazone-bound structure (ribbon in salmon) and lobeglitazone is shown by a green stick while the other ligands are shown in blue lines. Most ligands occupy the canonical LBP with Y-shaped pocket. The Cdk5-mediated phosphorylation site, Ser245, is represented by red sticks. The residues forming the hydrophobic pocket are shown in gray sticks.

The most distinctive structural difference between lobeglitazone and rosiglitazone is the *p*-methoxyphenol group that is elongated from the pyrimidine group of lobeglitazone (Fig. 1). Our structure of lobeglitazone-bound PPARγ LBD revealed that the *p*-methoxyphenol group of lobeglitazone occupies the hydrophobic pocket near the alternate binding site (Fig. 6a). This hydrophobic pocket corresponds to the third arm of canonical LBP of PPARγ^19^ and hydrophobic residues such as Ile249 (β1), Leu255 (H2’), Ile281 (H3), Ile341 (β3), and Met348 (β4) form this unique pocket. In addition, a conformational change of Arg280 occurs in the lobeglitazone-bound PPARγ LBD structure when compared with the rosiglitazone-bound structure, and the ether group in the *p*-methoxyphenol moiety of lobeglitazone forms a weak hydrogen bond with N_η_ atom of Arg280 (Fig. 6). When we superimposed the structures of many different ligands taken from 131 PPARγ LBD structures onto our lobeglitazone-bound PPARγ LBD structure, we observed that some ligands such as 5-substituted 2-benzoylaminobenzoic acids (2-BABAs) or amorfrutins occupy this hydrophobic pocket^25,26^ (Fig. 7). The ligand binding Gibbs free energies calculated from Autodock4 (The Scripps Research Institute, CA, USA)^27^ were −11.4 kcal/mol and −9.6 kcal/mol for lobeglitazone and rosiglitazone, respectively. The strong binding affinity of lobeglitazone is likely due to the tight binding of the *p*-methoxyphenol group in lobeglitazone to the hydrophobic pocket near the alternate binding site as well as the classical hydrogen bond networks between the TZD head group of lobeglitazone and AF-2 helix of PPARγ LBD.

### Lobeglitazone strongly blocks the phosphorylation of PPARγ at Ser245

**Figure 8:**
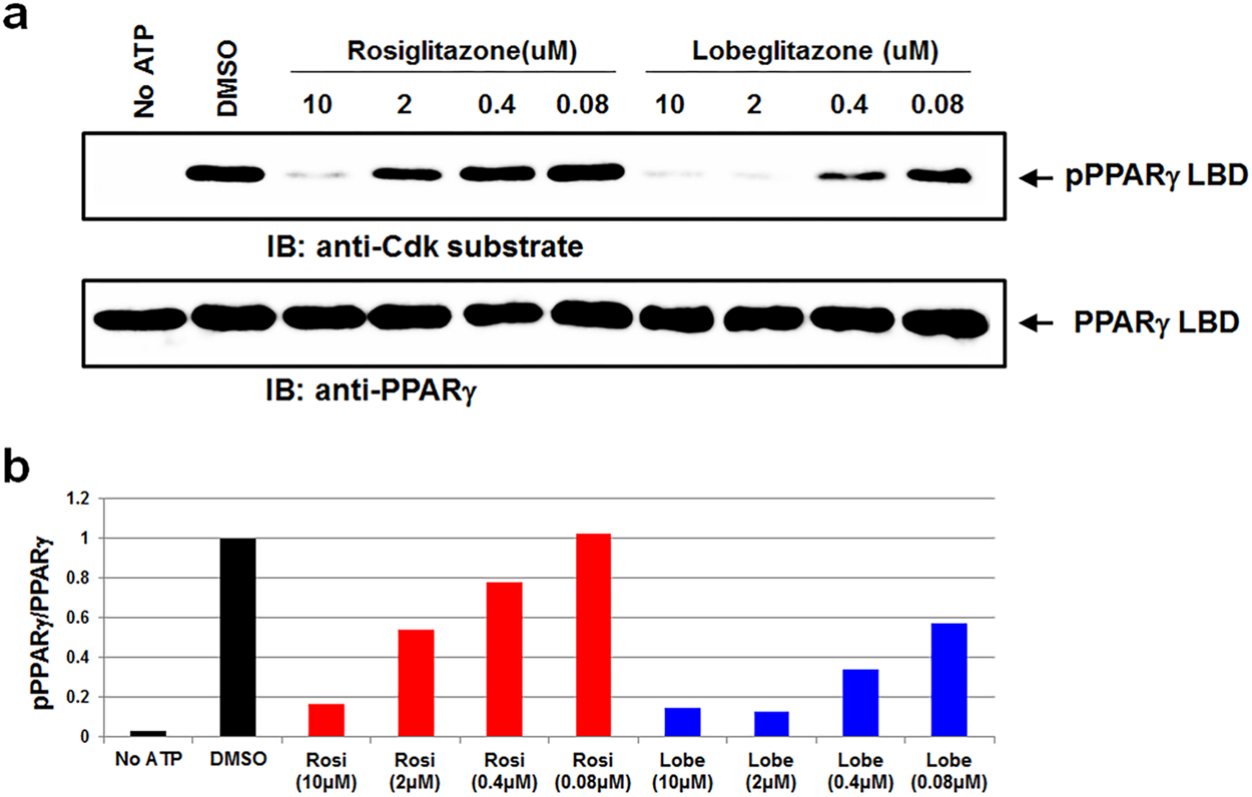
Inhibition of the Cdk5-mediated phosphorylation of PPARγ at Ser245 by lobeglitazone or rosiglitazone. (**a**) *In vitro* Cdk5 assay results with PPARγ LBD incubated with rosiglitazone or lobeglitazone in a dose-dependent manner. pPPARγ, phosphorylated PPARγ. (**b**) Quantification of PPARγ phosphorylation compared with total PPARγ *in vitro*. Rosi, rosiglitazone; Lobe, lobeglitazone.

Lobeglitazone showed 2.4-fold increase in glucose lowering activity in genetically diabetic KKA^y^ mice *in vivo* and 100 times increased efficacy in the enhancement of insulin-induced triglyceride accumulation in 3T3-L1 cells *in vitro*, compared with rosiglitazone^23^. However, it has not been well understood how lobeglitazone elicits more potent anti-diabetic effects in both *in vitro* and *in vivo* studies. Some of synthetic PPARγ ligands have been known to inhibit the phosphorylation of PPARγ at Ser245 and the post-translational modification is very important in anti-diabetic effects. Rosiglitazone also effectively blocked Cdk5-mediated phosphorylation of PPARγ at Ser245 *in vitro* ^18^. We performed an *in vitro* Cdk5 assay to determine whether lobeglitazone affects Cdk5-mediated phosphorylation of PPARγ at Ser245. Our result showed that lobeglitazone also blocks Cdk5-mediated phosphorylation of PPARγ at Ser245 *in vitro*, with half maximal inhibitory concentration of about 80 nM and lobeglitazone more potently inhibits the phosphorylation of PPARγ at Ser245 than rosiglitazone does (Fig. 8).

## Discussion

Lobeglitazone is a potent anti-diabetic drug with full agonist activity on PPARγ. In this study, we determined the structure of lobeglitazone-bound PPARγ LBD using X-ray crystallography. Rosiglitazone-bound PPARγ LBD structure was also determined for accurate comparative analysis. Lobeglitazone is structurally similar to rosiglitazone and belongs to a TZD drug. In lobeglitazone, the pyridine group of rosiglitazone is replaced with a pyrimidine group and the *p*-methoxyphenol moiety is added to the pyrimidine group (Fig. 1). The *p*-methoxyphenol group of lobeglitazone seems to be the most important part that is responsible for the improved binding affinity to PPARγ LBD compared with rosiglitazone. The TZD head group of both lobeglitazone and rosiglitazone forms strong hydrogen bond networks with AF-2 helix region (Ser289, His323, His449, and Tyr473) (Fig. 4c and Supplementary Fig. S1c). In addition to the TZD head group, the *p*-methoxyphenol group of lobeglitazone is further stabilized via hydrophobic effects from the hydrophobic pocket near the alternate binding site of PPARγ LBD (Fig. 6a). Moreover, the ether group in the *p*-methoxyphenol group of lobeglitazone forms a weak hydrogen bond with helix H3 of PPARγ LBD (Fig. 6a). *In vitro* kinase experiments showed that lobeglitazone more potently inhibits the Cdk5-mediated phosphorylation of PPARγ at Ser245 than rosiglitazone does (Fig. 8). Taken together, our results suggest that the additional interactions of *p*-methoxyphenol moiety in lobeglitazone with PPARγ LBD lead to stronger binding affinity than rosiglitazone and this enhanced affinity elicits a potent anti-diabetic effect accompanied with the increased inhibition of the Cdk5-mediated phosphorylation of PPARγ at Ser245.

When we compared C_α_ RMSD values between lobeglitazone-bound and rosiglitazone-bound PPARγ LBD structures, there was no remarkable structural differences among them (Fig. 3). Therefore, it seems difficult to understand the mechanism associated with the anti-diabetic effect from structural differences in lobeglitazone-bound and rosiglitazone-bound PPARγ LBD structures. However, when lobeglitazone-bound and rosiglitazone-bound PPARγ LDB structures were compared with the apo PPARγ LBD structure (PDB ID: 1PRG), the H2-β1 loop region where Ser245 is located, Ω loop region, and H11-H12 loop region show considerable conformational changes (Figs 2 and 3). The conformational changes in the H11-H12 loop region of PPARγ are presumably induced by the interaction of PPARγ with the TZD head group of lobeglitazone and rosiglitazone. This interaction affects the general transcription activity of PPARγ, as is well known^11^. The H2-β1 loop region includes Ser245, a residue of Cdk5-mediated phosphorylation, thus conformational changes in the H2-β1 loop could be related to the Cdk5-mediated phosphorylation of PPARγ. According to the *in silico* molecular recognition modeling for the PPARγ-Cdk5/p25 complex and for the protein–protein interactions between PPARγ and Cdk5/p25, the H2-β1 loop region containing Ser245, the Ω loop region, and the β-sheet site (residues Asn335, Lys336, Asp337, Thr349, and Glu351) of PPARγ would affect the binding of Cdk5 to PPARγ^28^. Our structural data also consistently show that binding of either lobeglitazone or rosiglitazone to PPARγ LBD induces large conformational changes in the H2-β1 loop region where Ser245 is located (Figs 2 and 3).

In addition, Choi *et al*. proposed that ligand binding reduces dynamic natures of helix H3 (amino acids 281–287), the β-sheet region (amino acids 341–351), and the Cdk5 site in the H2-β1 loop region of PPARγ and ligand-bound PPARγ adopts a less favorable configuration for the Cdk5-mediated phosphorylation of PPARγ^18,20^. To elucidate the lobeglitazone-induced structural stability of PPARγ, we compared normalized B-factors in the crystal structures of lobeglitazone-bound, rosiglitazone-bound, apo PPARγ LDB in complex with SRC-1 (PDB ID: 5GTP), and apo PPARγ LBD (PDB ID: 1PRG) (Supplementary Fig. S2). Our B-factor analysis also supported that binding of either lobeglitazone or rosiglitazone to PPARγ LBD stabilizes helix H3. However, we could not observe noticeable B-factor changes in the Cdk5 site of PPARγ, which is consistent with previously reported HDX data with rosiglitazone^18^, and in the β-sheet region (amino acids 341–351). Consequently, the binding of TZD drugs such as lobeglitazone and rosiglitazone causes structural changes in the PPARγ motif for the interaction with Cdk5 and the binding of Cdk5 is weakened, resulting in the suppression of the Cdk5-mediated phosphorylation of PPARγ at Ser245.

*In silico* drug design approaches are commonly used for the target identification, validation, molecular design, and drug interactions with target proteins^29^. A number of computer-aided drug discovery studies have been also conducted to find new insulin sensitizing molecules targeting PPARγ with reduced toxicity and side effects^30^. *In silico* studies have been reported using the structure of PPARγ LBD in complex with rosiglitazone, a representative anti-diabetic drug for PPARγ^31,32^. Thus, the exact binding mode of rosiglitazone to PPARγ LBD is of great importance to ensure reliable results. We found that there is a slight structural difference in the methylamino group of rosiglitazone in our PPARγ LBD structure and in the representative PPARγ LBD structure with PDB ID 2PRG^11^ (Fig. 5). To determine the exact conformation of the methylamino group of rosiglitazone, we superimposed all the known PPARγ LBD structures in complex with rosiglitazone (PDB IDs: 1FM6, 2PRG, 3CS8, 3DZY, 4EMA, 4O8F, and 4XLD)^11,33–38^. Superposition of all the rosiglitazone-bound PPARγ LBD structures showed that the methylamino group in the PPARγ LBD structure with PDB ID 2PRG is solely directed upward and all the other methylamino groups in rosiglitazone are directed downward with respect to helix H3 (Supplementary Fig. S3). Based on the current structural information summarized by our study, we suggest that the downward conformation of the methylamino group in rosiglitazone and other structurally similar TZD drugs, with respect to helix H3, needs to be firstly considered for *in silico* studies in the development of new anti-diabetic drugs with PPARγ.

Many derivatives of thiazolidinedione were tested at the drug development stage. In the case of lobeglitazone, the pyrimidine group was substituted for the pyridine group of rosiglitazone and the *p*-methoxyphenol group was added at the 4-position of the pyrimidine moiety (Fig. 1). Lobeglitazone with the *p*-methoxyphenol group showed a much better efficacy in the insulin-regulated differentiation with 3T3-L1 cells than other 4-position substituted pyrimidine derivatives with *iso*-propoxy group, phenylamino group, and so on^23^. From our structural analysis, the *p*-methoxyphenol group in lobeglitazone could tightly occupy the hydrophobic pocket of PPARγ LBD and it would explain the enhanced biological activities of lobeglitazone (Fig. 6). It is also worth noting that the *p*-methoxyphenol group of lobeglitazone is stabilized by the hydrophobic pocket near the alternate binding site. Many synthetic ligands such as 2-BABAs and amorfrutins have been reported to bind to this hydrophobic pocket of PPARγ (Fig. 7). Interestingly, both 2-BABAs and amorfrutins are known to be selective PPARγ modulators (SPPARγMs) that regulate PPARγ without direct interaction with AF-2 helix^25,26^. The selective PPARγ modulation is a new pharmacological approach that produces a potent anti-diabetic effect based on selective receptor-ligand interaction and target gene regulation, minimizing the side effects associated with PPARγ^39^. Considering the characteristics of PPARγ ligands that occupy the hydrophobic pocket, this hydrophobic pocket seems to be closely related to SPPARγM. It has been also reported that new synthetic ligands that bind to the alternate binding site effectively block the Cdk5-mediated phosphorylation of PPARγ at Ser245 and have potent anti-diabetic effects^19,21^. Thus, we could anticipate the emergence of new anti-diabetic agents with well reduced side effects if we pay more attention to the hydrophobic pocket and the alternate binding site of PPARγ LBD.

In conclusion, we have showed that lobeglitazone binds to PPARγ LBD more strongly than rosiglitazone does due to the additional *p*-methoxyphenol group. Even though we could not clearly correlate anti-diabetic effects by lobeglitazone or rosiglitazone with structural differences in lobeglitazone-bound or rosiglitazone-bound PPARγ, we showed that lobeglitazone induces a conformational change in the H2-β1 loop where Ser245 is located, which could inhibit the Cdk5-mediated phosphorylation of PPARγ at Ser245 even at a lower concentration than rosiglitazone did *in vitro*. These observations explain the enhanced anti-diabetic efficacy of lobeglitazone derived from tighter ligand binding to PPARγ LBD. It still needs further investigation whether the enhanced efficacy of lobeglitazone could mitigate many of the known side effects of the TZD drugs and what would be the underlying mechanism for the increased inhibition of Cdk5-mediated phosphorylation of PPARγ at Ser245 by lobeglitazone, compared with the inhibition by rosiglitazone. Thus, to develop better PPARγ-targeting drugs, comprehensive understanding is essential, including anti-diabetic effects, Cdk5-mediated phosphorylation of PPARγ at Ser245, PPARγ agonism, side effects after administration of drugs, and so-far unclear mechanisms of endogenous ligands. We still need more information about therapeutic benefits and structures of PPARγ in complex with ligands eliciting full, partial, or no agonism. Our study provides an insight on the structure-based discovery of new PPARγ ligands as an anti-diabetic drug minimizing known side effects.

## Methods

### Protein expression and purification

Human PPARγ LBD construct (residues 195–477 in PPARγ1 numbering) was PCR-amplified from a human cDNA clone encoding PPARγ (clone ID: hMU000317) as the template, which was purchased from the Korea Human Gene Bank, Medical Genomics Research Center, KRIBB and cloned into the expression vector pET-28b(+) (Novagen). The recombinant human PPARγ LBD protein has a 21-residue N-terminal fusion tag (MGSSHHHHHH SSGLVPRGSHM) containing a His_6_ tag and a thrombin cleavage site in front of the starting residue Ala195. The protein was overexpressed in *Escherichia coli* Rosetta 2(DE3) cells using the Luria-Bertani medium that contained 30 μg/mL kanamycin. Human PPARγ LBD protein expression was induced by 0.5 mM isopropyl β-d-thiogalactopyranoside and the cells were incubated for additional 20 h at 18 °C following growth to mid-log phase at 37 °C. The cells were lysed by sonication in buffer A (20 mM Tris-HCl at pH 8.5, 150 mM NaCl, 10% (v/v) glycerol and 0.1 mM tris(2-carboxyethyl) phosphine hydrochloride) containing 5 mM imidazole and 1 mM phenylmethylsulfonyl fluoride. The crude lysate was centrifuged at 36,000 × g for 50 min at 4 °C. The supernatant was applied to an affinity chromatography column of HiTrap Chelating HP (GE Healthcare), which was previously equilibrated with buffer A containing 5 mM imidazole. The recombinant human PPARγ LBD was eluted at 50–100 mM imidazole concentration, upon applying a gradient of imidazole in the same buffer. The eluted protein was de-salted in buffer A using a desalting column of HiPrep 26/10 (GE Healthcare) to remove imidazole, and the protein was cleaved with 2 units of thrombin (Merck Millipore) per mg of PPARγ LBD at 4 °C overnight. Both the N-terminal fusion tag and the uncleaved protein were removed by affinity chromatography on a HiTrap Chelating HP column. The flow-through was applied to a gel filtration chromatography column of HiLoad XK-16 Superdex 200 prep-grade (GE Healthcare), which was previously equilibrated with buffer A. Fractions containing the human PPARγ LBD were pooled and concentrated to 15.8 mg/mL using an Amicon Ultra-15 Centrifugal Filter Unit (Merck Millipore).

### Crystallization

Before crystallization, the purified PPARγ LBD and the LXXLL motif-containing peptide (ERHKILHRLLQEGSPS corresponding to residues 685–700 of the human SRC-1) were mixed in a molar ratio of 1:2, in the presence of a 7-fold molar excess of the PPARγ ligand lobeglitazone or rosiglitazone. After an overnight incubation, the protein-ligand complexes were crystallized by the sitting-drop vapor diffusion method using the Mosquito robotic system (TTP Labtech) at 23 °C by mixing 0.2 μl of the protein solution and 0.2 μl of the reservoir solution. Crystals of PPARγ·Lobeglitazone·SRC-1 were obtained with a reservoir solution of 1.6 M sodium citrate tribasic dihydrate at pH 6.5. PPARγ·Rosiglitazone·SRC-1 crystals were obtained with a reservoir solution of 2.2 M sodium malonate at pH 7.0. In both cases, the initial crystals appeared as multiple crystals that were not suitable for diffraction data collection. Therefore, microseeding technique was used to obtain single crystals. Several pieces of the initial crystals were transferred into an Eppendorf tube containing a Seed Bead (Hampton Research) and 50 μl reservoir solution and crystals were vortexed to produce microseeds. The stock solution of microseeds was then briefly centrifuged and diluted serially by a factor of 100–1000 in the same reservoir solution. Each sitting drop was prepared by mixing the protein solution, the reservoir solution, and the microseed solution in a volume ratio of 1:0.7:0.3. Single crystals of PPARγ·Lobeglitazone·SRC-1 and PPARγ·Rosiglitazone·SRC-1 grew reproducibly to dimensions of approximately 0.35 × 0.2 × 0.1 mm and 0.2 × 0.2 × 0.1 mm, respectively, within a few days.

### X-ray data collection

X-ray diffraction data for lobeglitazone-bound PPARγ LBD were collected at 100 K using a Quantum Q270 CCD detector system (Area Detector Systems Corporation, Poway, California) at the BL-7A experimental station of Pohang Light Source, Korea. The X-ray data from the crystal of rosiglitazone-bound PPARγ LBD were collected at 100 K using a Quantum 315r CCD detector system (Area Detector Systems Corporation, Poway, California) at the BL-5C experimental station of Pohang Light Source, Korea. Raw X-ray diffraction data were processed and scaled using the program suit *HKL2000* ^40^. Crystals of lobeglitazone-bound PPARγ LBD belong to the space group *P2*_1_2_1_2, with unit cell parameters of a = 131.0 Å, b = 53.2 Å, c = 54.9 Å. One monomer is present in the asymmetric unit, giving a Matthew’s parameter and solvent fraction of 2.75 Å^3^ Da^−1^ and 55.3%, respectively. Crystals of rosiglitazone-bound PPARγ LBD belong to the space group *P2*_1_2_1_2, with unit cell parameters of a = 130.8 Å, b = 53.1 Å, c = 54.6 Å. One monomer is present in the asymmetric unit, giving a Matthew’s parameter and solvent fraction of 2.75 Å^3^ Da^−1^ and 55.3%, respectively. Data collection statistics are summarized in Supplementary Table S1.

### Structure determination and refinement

Both structures were solved by molecular replacement method with the program *MolRep* ^41^ using the previously published PPARγ LBD structure (PDB ID: 5GTO)^19^ as a search model. Subsequent model building was done manually using the program *COOT* ^42^ and the models were refined with the program *REFMAC5* ^43^, including the bulk solvent correction. A total of 5% of the data was randomly set aside as test data for the calculation of *R*_*free*_^44^. The stereochemistry of the refined models was assessed by *MolProbity* ^45^. Water molecules were added using the program *COOT* ^42^, followed by visual inspection and B-factor refinement. Refinement statistics are summarized in Supplementary Table S1.

### *In vitro* kinase assay

*In vitro* Cdk5 assay was conducted according to the manufacturer’s instruction (Cell Signaling Technology). In Brief, 0.5 μg of the purified PPARγ LBD was incubated with active Cdk5/p35 (Millipore) in assay buffer (25 mM Tris-HCl at pH 7.5, 5 mM β-glycerophosphate, 2 mM dithiothreitol, 0.1 mM Na_3_VO_4_, 10 mM MgCl_2_) containing 25 μM ATP for 30 min at 30 °C. Various concentrations of lobeglitazone and rosiglitazone ranging from 0.08 to 10 μM were pre-incubated with substrates for 30 min at 30 °C before performing the kinase assay. Phosphorylation by Cdk5 was analyzed by Western blotting using an anti-Cdk substrate antibody to detect phospho-Ser in a [K/R]-S-P-X-[K/R] motif (Cell Signaling Technology).

### Accession codes

Atomic coordinates and structure factors have been deposited in Protein Data Bank under the accession codes 5YCN and 5YCP for the lobeglitazone-bound and rosiglitazone-bound structures, respectively.

## Electronic supplementary material

Supplementary Information
