# Supplementary material for: Structural Basis for the Enhanced Anti-Diabetic Efficacy of Lobeglitazone on PPARγ
Source: Sci Rep. 2018 Jan 8;8:31. doi: 10.1038/s41598-017-18274-1 (PMC5758645; doi:10.1038/s41598-017-18274-1)

Supplementary Data

Structural Basis for the Enhanced Anti-Diabetic Efficacy of Lobeglitazone on PPARγ

Jun Young Jang[1], Hwan Bae[2], Yong Jae Lee[3], Young Il Choi[3], Hyun-Jung Kim[4], Seung Bum Park[2], Se Won Suh[2], Sang Wan Kim[5*], and Byung Woo Han[1*]

[1]Research Institute of Pharmaceutical Sciences, College of Pharmacy, Seoul National University, Seoul 08826, Republic of Korea

[2]Department of Chemistry, College of Natural Sciences, Seoul National University, Seoul 08826, Republic of Korea

[3]CKD Research Institute, Chong Kun Dang Pharmaceutical Corporation, Yongin 16995, Republic of Korea

[4]College of Pharmacy, Chung-Ang University, Seoul 06974, Republic of Korea

[5]College of Medicine, Seoul National University, Seoul 03080, Republic of Korea

*Correspondence and requests for materials should be addressed to S.W.K. (email: swkimmd@snu.ac.kr) or B.W.H. (bwhan@snu.ac.kr).

Supplementary Table S1. Statistics for the data collection and model refinement.

Model name	Lobeglitazone-bound PPARγ LDB	Rosiglitazone-bound PPARγ LBD
A. Data collection		
X-ray source	PLS-7A	PLS-5C
X-ray wavelength (Å)	0.97935	0.97960
Space group	$P2_12_12$	$P2_12_12$
Unit cell parameters		
a (Å)	130.96	130.81
b (Å)	53.17	53.13
c (Å)	54.86	54.57
$\alpha = \beta = \gamma$ (°)	90	90
Resolution range (Å)	50.0–2.15 (2.19–2.15)	50.0–2.00 (2.03–2.00)
Total / unique reflections	97,957 / 21,669	262,323 / 26,589
Completeness (%)	99.8 (100.0)	99.9 (100.0)
$<I / \sigma_I>$	31.0 (3.6)	53.4 (5.4)
R_{merge} (%)	6.8 (51.4)	6.4 (59.1)
$CC_{1/2}$	0.962 (0.835)	0.988 (0.986)
B. Model refinement		
Resolution range (Å)	30.0–2.15	30.0–2.00
R_{work} / R_{free} (%)	20.8 / 22.8	20.3 / 23.3
No. of non-hydrogen atoms		
Protein	2206	2206
Ligand	34	25
Water oxygen	113	85
Average B factor (Å^2)		
Protein	46.7	44.6
Ligand	40.0	35.9
Water oxygen	49.1	44.8
R.m.s. deviations from ideal geometry		
Bond lengths (Å)	0.010	0.012
Bond angles (°)	1.49	1.49
Ramachandran plot		
Favored / Outliers (%)	98.5 / 0.0	98.1 / 0.0
Poor rotamers (%)	0.00	0.00
Values in parentheses refer to the highest resolution shell.		

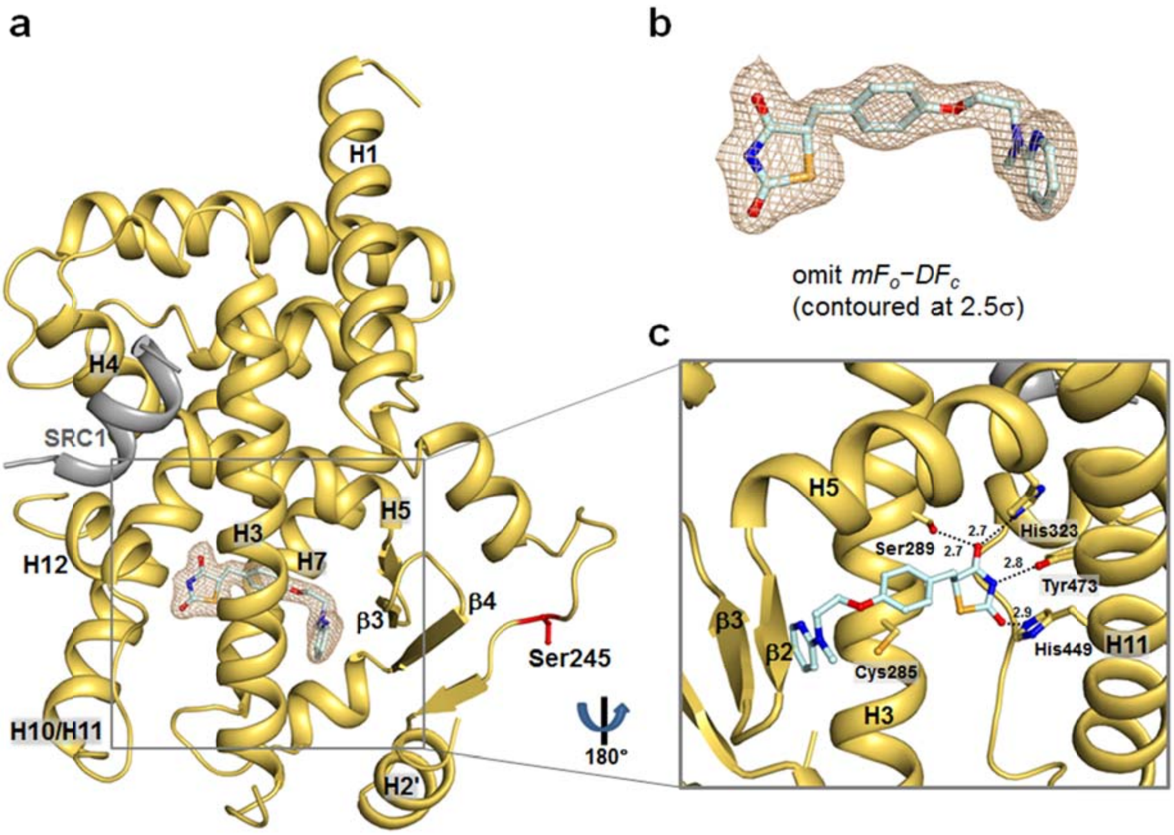

Supplementary Figure S1. Overall structure of rosiglitazone-bound PPARγ LBD. (a) Ribbon diagram of rosiglitazone-bound PPARγ LBD (yellow orange) with the SRC-1 coactivator peptide (gray). Rosiglitazone shown as a pale cyan stick model occupies the LBP of PPARγ. The electron density for rosiglitazone in the *mFo-DFc* omit map is shown as a wheat-colored mesh (contoured at 2.5σ). The Cdk5-mediated phosphorylation site, Ser245, is represented by red sticks. (b) Close-up view of bound rosiglitazone in sticks with the *mFo-DFc* omit electron density map (contoured at 2.5σ). (c) Close-up view of interactions between PPARγ LBD and rosiglitazone. The view is 180° rotated from the orientation in (a). Hydrogen bonds are depicted by dashed black lines and labeled with donor–acceptor

distances in Å. Helix H7 has been omitted to show clear position of rosiglitazone. Cys285 is represented in sticks.

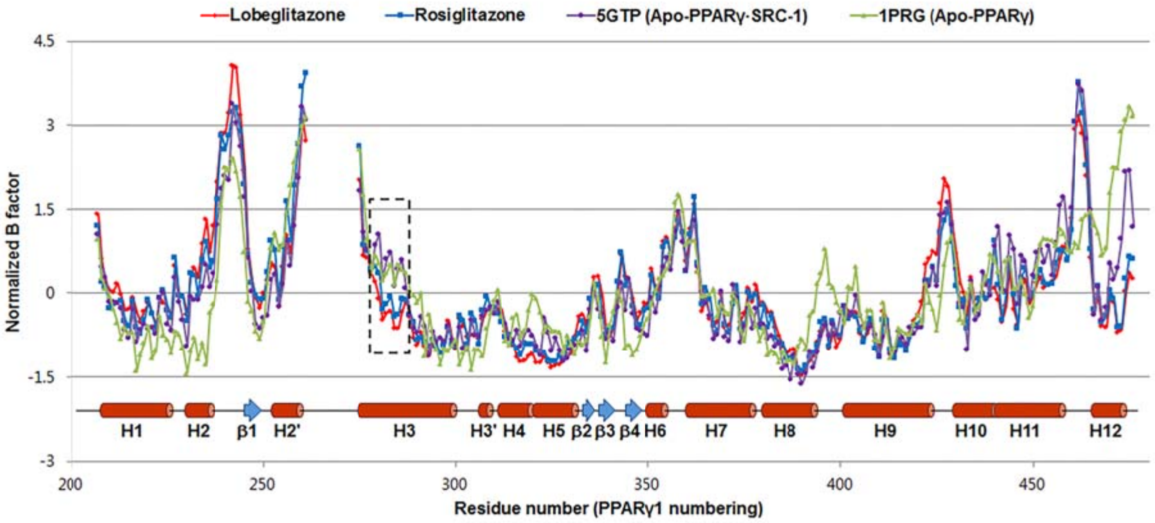

Supplementary Figure S2. Comparison of normalized B-factors of PPARγ structures. B-factors of C_α atoms for the lobeglitazone-bound, rosiglitazone-bound, apo PPARγ LDB in complex with SRC-1 (PDB ID: 5GTP), and apo PPARγ LBD (PDB ID: 1PRG) were compared. Binding of either lobeglitazone or rosiglitazone to PPARγ LBD stabilizes helix H3. Helix H3 region with decreased B-factors upon ligand-binding is indicated by black dashed rectangle. Secondary structure elements are indicated on the residue numbers.

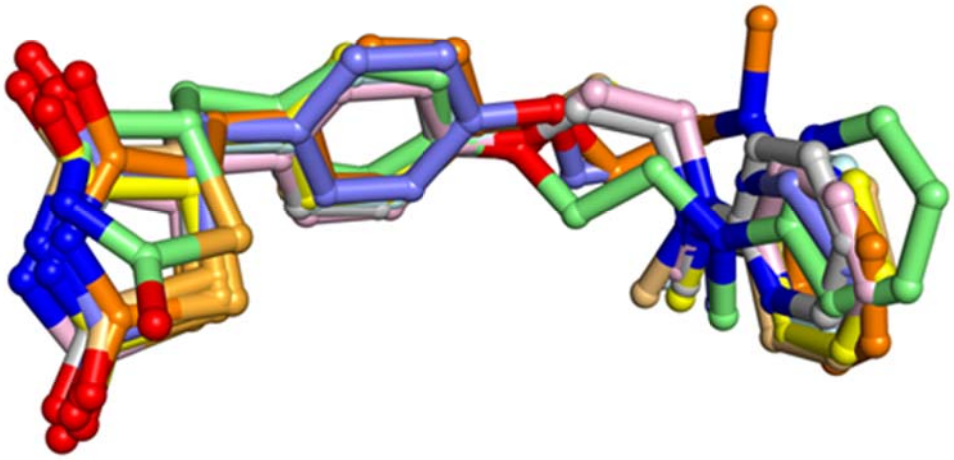

Supplementary Figure S3. Comparison of binding modes of rosiglitazones to PPARγ LBD. Superposition of rosiglitazones taken from our rosiglitazone-bound PPARγ LBD structure (colored in pale cyan) and from previously reported rosiglitazone-bound PPARγ LBD structures reveals that the methylamino group of rosiglitazone is directed downward with respect to helix H3, except the structure with PDB ID 2PRG. PDB IDs and represented colors are as follows: 1FM6, yellow; 2PRG, orange; 3CS8, lime; 3DZY, light orange; 4EMA, gray; 4O8F, slate; 4XLD, light pink.

Full length blots used in the main figure

Figure 8. a

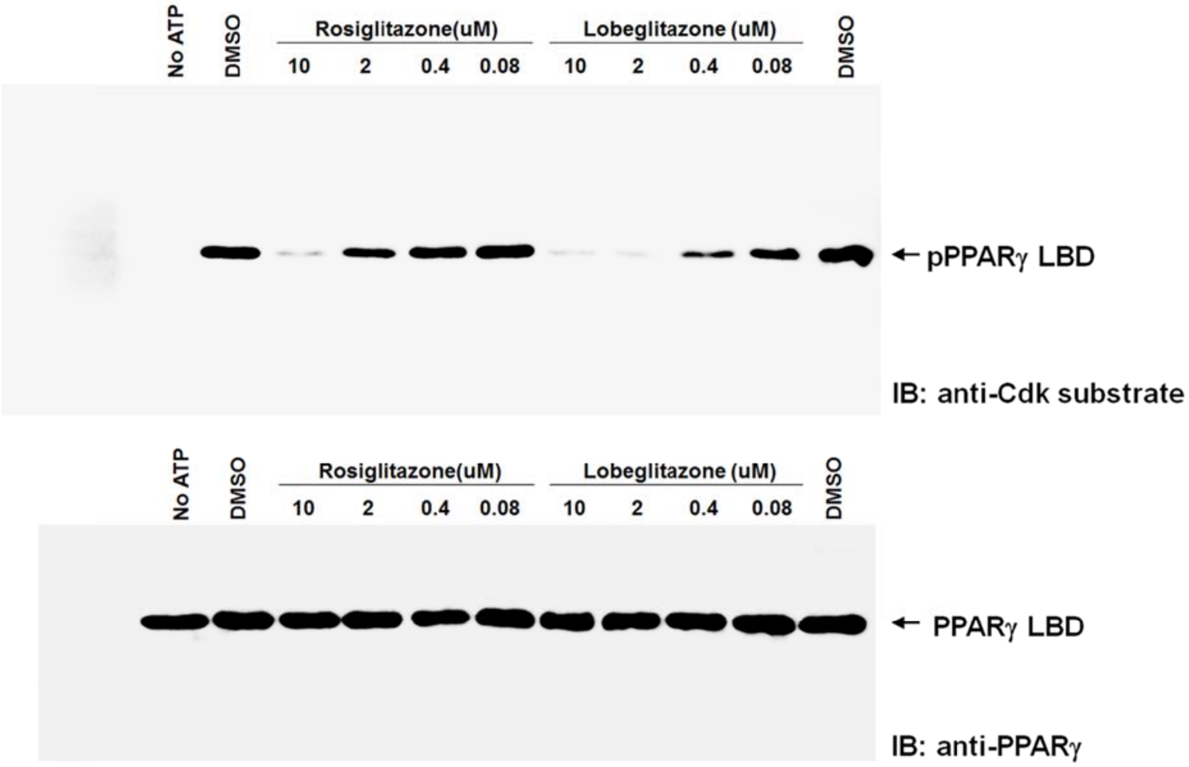

Supplement: Supplementary file 1 — Supplementary Information [file 41598_2017_18274_MOESM1_ESM.pdf]
